# Supplementary material for: Toxoplasma induced cytokine release syndrome is critically dependent on the expression of pore-forming Perforin-Like Protein-1
Source: bioRxiv. 2025 Mar 17:2025.03.17.643671. Preprint. [Version 1] doi: 10.1101/2025.03.17.643671 (PMC11956978; doi:10.1101/2025.03.17.643671)
Supplement: 1 [file NIHPP2025.03.17.643671V1-supplement-1.pdf]

bioRxiv preprint doi: https://doi.org/10.1101/2025.03.17.643671; this version posted March 17, 2025. The copyright holder for this preprint (which was not certified by peer review) is the author/funder. This article is a US Government work. It is not subject to copyright under 17 USC 105 and is also made available for use under a CC0 license.

Supporting Information

S1 Fig. Gating strategy for flow cytometric analysis. PEC immune cell populations referenced in figures 4 and 5, were identified based on sequential phenotyping by immunological markers. PECs were gated based on FSC-A/SSC-A (A) followed by doublet exclusion (B - singlets, FSC-A/FSC-H). The live cell population was delineated by low levels of LiveDead Fixable Blue staining (C) and hematopoetic cells were gated as $CD45^+$ (E). From the $CD45^+$ cells, neutrophils were gated as $CD11b^-$/$Ly6G^+$ (F) and the $CD11b^+$ cell population was further divided by expression of CD11c (G). $CD11c^+$ cells were confirmed as dendritic cells by expression of MHC-II (H), while $CD11c^-$ cells were

bioRxiv preprint doi: https://doi.org/10.1101/2025.03.17.643671; this version posted March 17, 2025. The copyright holder for this preprint (which was not certified by peer review) is the author/funder. This article is a US Government work. It is not subject to copyright under 17 USC 105 and is also made available for use under a CC0 license.

phenotyped as either F4/80^{+} macrophages or F4/80^{-}/Ly6C^{+} monocytes (I). Total *Toxoplasma* infected PECs were accounted for by GFP expression in the CD45^{+} cell population (J). Diagram is a representative example from the WT RH infected mice 4 dpi.

S2 Fig. RH PLP1 knockout parasites induce cytokine and chemokine responses after infection. PEC lavage (A-E) and serum (F-J) cytokine and chemokine responses were measured and graphed for 10^4 *Δplp1* (green), or 10^6 *Δplp1* (blue) i.p. infection of C57BL/6 mice. IL-6 (A, F), IFN-g (B, G), MCP-1/CCL2 (C, H), MIP1-b/CCL4 (D, I), and G-CSF (E, J), were assessed by Milliplex between days 3 and 11 post-infection, as described. Graphs are representative results from one of two experiments (n=3 with a single PBS control per day). Data for RH infection is omitted to better demonstrate changes induced by PLP1 deficient parasite infection. Two-way ANOVA statistical analysis was used to determine significance; ** represents $p \leq 0.01$, *** represents $p \leq 0.001$.

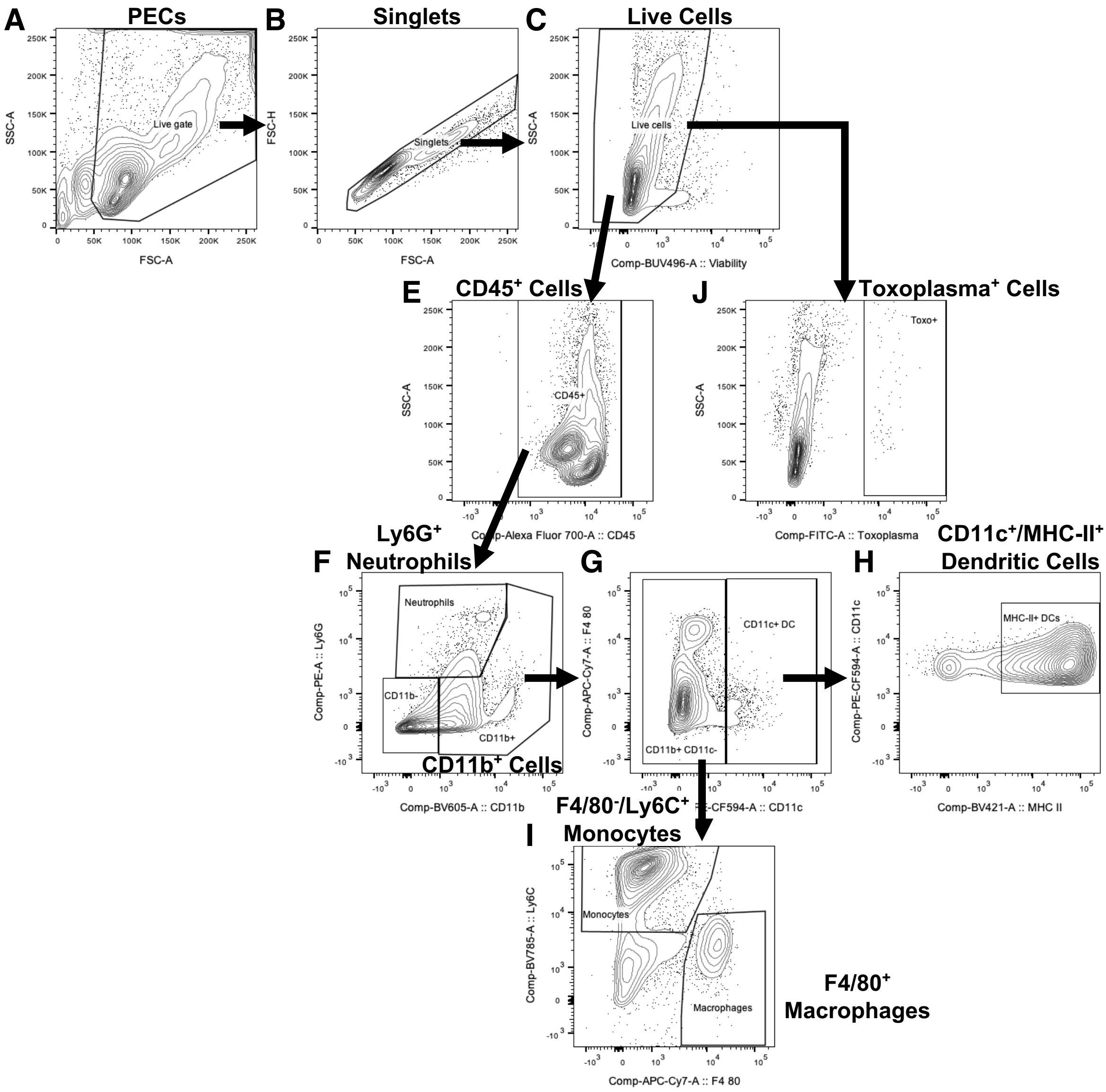
A
PECs
Live gate
SSC-A
FSC-A
B
Singlets
Singlets
FSC-H
FSC-A
C
Live Cells
Live cells
SSC-A
Comp-BUV496-A :: Viability
E
CD45+ Cells
CD45+
SSC-A
Comp-Alexa Fluor 700-A :: CD45
J
Toxoplasma+ Cells
Toxo+
SSC-A
Comp-FITC-A :: Toxoplasma
F
Ly6G+ Neutrophils
Neutrophils
CD11b-
CD11b+
CD11b+ Cells
Comp-PE-A :: Ly6G
Comp-BV605-A :: CD11b
G
CD11c+ DC
CD11b+ CD11c-
Comp-APC-Cy7-A :: F4 80
H
CD11c+/MHC-II+ Dendritic Cells
MHC-II+ DCs
Comp-PE-CF594-A :: CD11c
Comp-BV421-A :: MHC II
I
F4/80-/Ly6C+ Monocytes
Monocytes
Macrophages
Comp-BV785-A :: Ly6C
Comp-APC-Cy7-A :: F4 80
F4/80+ Macrophages

S1 Fig

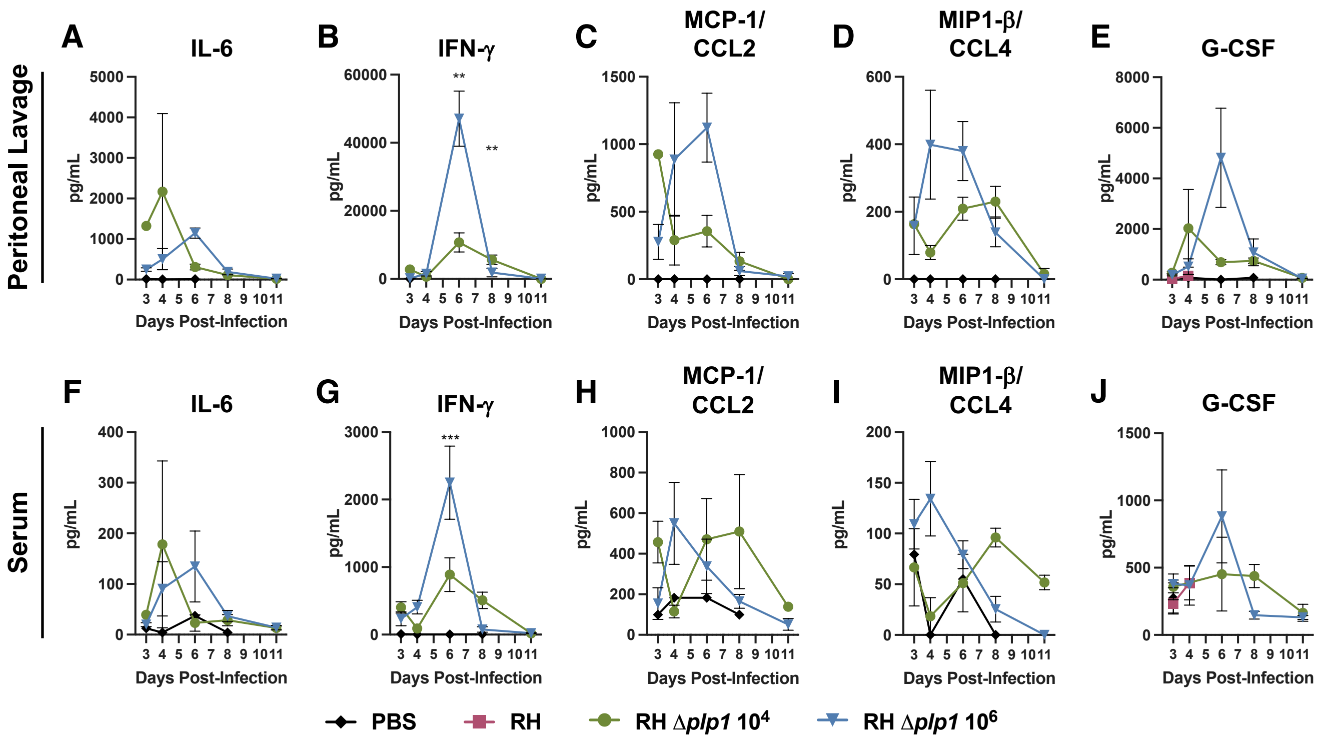

A
IL-6
B
IFN-γ
C
MCP-1/
CCL2
D
MIP1-β/
CCL4
E
G-CSF
Peritoneal Lavage
Serum
F
IL-6
G
IFN-γ
H
MCP-1/
CCL2
I
MIP1-β/
CCL4
J
G-CSF
pg/mL
Days Post-Infection
PBS
RH
RH Δplp1 10^4
RH Δplp1 10^6

S2 Fig
